# A customer value analysis of Taiwan ice cream market: a means-end chain approach across consumption situations

**DOI:** 10.1186/s40064-015-1564-8

**Published:** 2015-12-08

**Authors:** Yan-Kwang Chen, Pei-Shan Tsai, Fei-Rung Chiu

**Affiliations:** Department of Distribution Management, National Taichung University of Science and Technology, 129 Sanmin Road, Sec. 3, Taichung, Taiwan; Department of Tourism and Recreation Management, Overseas Chinese University, 100,Chiao Kwang Rd., Taichung, 40721 Taiwan

**Keywords:** Ice cream, Means-end chain, Implication matrix, Hierarchical value map, Consumption situation

## Abstract

In the highly competitive market, it is increasingly hard for ice cream stores to develop creative marketing strategies to retain existing customers and attract new ones. This study applies the means–end chain approach to identify the customer value, consequences, and attributes of ice cream and to suggest useful information for ice cream sellers to develop differential marketing strategies across various consumption situations (i.e. on a date, gathering with friends, craving for ice cream). This study conducted one-on-one in-depth interviews with participants. The interview content was subsequently analyzed and coded to produce an implication matrix and a hierarchical value map, which was further used to determine customers’ value perceptions. The results indicate the terminal values of the highest strength comprised economy, pleasure, and efficiency. Pleasure was emphasized among consumers who were on a date or gathering with friends, whereas satisfaction was emphasized among consumers who craved ice cream. Based on the results, the study also provides suggestions to the industry and future researchers.

## Background

According to Customs Administration, Ministry of Finance of Taiwan, the volume of ice cream imports has increased 56 % over the last decade, from 3399 tons in 2005 to 5316 tons in 2014. This lucrative business opportunity has intensified the competition in the ice cream market, motivating all ice cream stores to create uniqueness in their products to differentiate their market or use marketing strategies to attract new customers. For instance, Cold Stone allows customers to create their ice cream mix using a wide variety of cookies, nuts, Brownie cakes, and other ingredients with as many as 12000 combinations. Haagen-Dazs offers seasonal products and festival-themed products, such as ice cream hot pot and ice cream moon cake. Bigtom has its marketing focus placed on the low-fat and healthy nature of its ice cream. Movenpick claims to be dedicated to 100 % customer satisfaction by providing 100 % natural ice cream.

Traditional marketing focuses more on product functionality, because it assumes customers to be rational decision makers who care about functional features and benefits (Holbrook 1999). With the rise of the experience economy, customers have begun to seek spiritual satisfaction while in pursuit of material satisfaction. Customers pay attention to the dining environment and aspire to gain a unique experience while enjoying the great food of a restaurant. Therefore, customers are no longer rational decision-makers. Instead, they are emotional decision-makers whose preferences may constantly change.

Providing an emotional experience for consumers has become a way to create a competitive advantage. To deepen customers’ impressions, many businesses integrate story elements into their advertisements. For instance, a Taiwan High Speed Rail advertisement featuring a father buying fish in the market depicts parents’ longing to see their children; the Barley Black Tea—Youth Book edition is another story-based advertisement reminding consumers of the feeling of sharing joy with friends. By telling a story, these advertisements are intended to involve customers in a context where they can interact with the advertised product. Using consumption situational factors, businesses can create a successful experiential service for customers. This means consumption situational factors are positively related to consumers’ behavioral intention. A good consumption situation can stimulate impulse buying and increase customer loyalty, which in turn can lead to higher business performance (Dong and Siu 2013). Therefore, creating an all-encompassing consumption situation is a way to influence customer decision-making.

Customers make purchase decisions based on their personal values. Therefore, customer value is viewed as a key predictor of customer behavior. A positive customer value can promote buying behavior, whereas a negative customer value may have a negative effect on buying behavior. High customer value is one of the main drivers of customers’ repatronage (Flint et al. 1997). However, the consumer decision-making process is a black-box mechanism, making it difficult for sellers to understand consumers’ true motivations, simply through tracking purchases or observing buying behaviors (Schmitt 1999).

The existing literature has focused on either the effects of consumer value/motivation or the effects of product attributes on consumer decision. Little literature has investigated the effects of both (Harris and Mitchell 2005). To increase profits, companies need to develop more effective marketing strategies. The first step in this process requires investigating customer demand value. Then, seek out the marketing mix of key product attributes that meet customer value to help retain existing customers and attract new customers (Lin and Chang 2012).

The use of a questionnaire survey in quantitative research has been noted to be unable to provide comprehensive insights into the relationship between consumer motivations and product attributes (Kahle and Kennedy 1989). Previous research employed the means-end chain (MEC) approach to explore how a person selects products or services that enable him or her to achieve the desired end state (Mattila 1999; Mosquera and Sánchez 2011). In this approach, “means” refers to the items to choose from, such as products or services, and “ends” are the values that one can get from their choice, such as happiness, security or achievement. MEC has been widely applied to new product development, brand image positioning, planning of advertising appeals, and market segmentation. This study builds MEC to investigate customer value in different consumption situations and provide marketing strategies that can better meet customers’ actual needs and ultimate goals.

We will use the fast-growing ice cream market as an example. Based on the means-end chain theory, we will conduct laddering interviews and draw hierarchical value maps (HVM) to explore the hierarchical relationship of value perceptions, consequences of consumption, and product attributes based on customer experiences of buying ice cream and further compare customer value between different consumption situations. The results can provide an insight into the relations between customer value hierarchies and help ice cream stores understand consumers’ actual needs and ultimate goals to offer services of higher customer value.

## Literature review

### Means-end chain

The means-end chain approach was introduced by Gutman (1982). This approach assumes consumers will identify the attributes of each product or service of their choice, the attributes influence their actual experience with the product or service, and their actual experience will further reinforce their desired value. This hierarchical structure of attribute-consequence-value can be used as an instrument for understanding consumers’ decision-making regarding the choice of a product or service to help them achieve their desired end state.

Olson and Reynolds (1983) proposed a model where attributes are divided into concrete attributes and abstract attributes, consequences are divided into functional consequences and psychosocial consequences, and values are divided into instrumental values and terminal values.

1. Attributes
  Attributes are the characteristics of a product or service. Generally, they can be classified into concrete attributes and abstract attributes. Concrete attributes are the tangible and objective aspects of a product that can be directly measured, experienced by touch or perceived by eye. Color, size, and price are attributes of this kind. Abstract attributes are the intangible and subjective aspects of a product, such as services and reputation, which are determined subjectively or by individual feeling. Kotler and Armstrong (1999) mentioned consumers view every product as a collective of some attributes. By choosing preferred attributes, consumers can obtain the desired consequences. Therefore, attributes are the cornerstone of the means-end chain.
2. Consequences
  At the center of the hierarchical structure, consequences connect products attributes to personal values. They are the direct or indirect results of consumption at the physical or mental level. If the results of consumption are positive, the results are called benefits; if the results of consumption are negative, the results are called risks. Levitt (1960) argues when customers mull over buying a product or brand, they think of the consequences rather than the attributes of the product or the brand. Olson and Reynolds (1983) identify two kinds of consequences, namely functional consequences and psychosocial consequences. Functional consequences refer to the instant physiological results of using a product. For instance, satisfying hunger is a functional consequence of cookies. Psychosocial consequences include psychological consequences and social consequences. Psychological consequences refer to the psychological effects of using a product, which can strengthen one’s image and dignity. For instance, one may feel more attractive and confident after using the perfume carrying a name brand. Social consequences refer to the results of interacting with others, such as an ideal image or a better social status. For instance, one may feel respect or admiration when he or she buys an expensive sedan. Consequences can satisfy personal values and goals (Gutman 1982; Reynolds et al. 2001; Parry 2002).
3. Values
  In the means-end chain theory, values are defined as the desired end state (Gutman 1982). Values are related to one’s life goals and satisfaction of individual needs (Peter and Olson 1996). Kahle and Timmer (1983) developed a list of values (LOV) based on Maslow (1954) hierarchy of needs and Rokeach (1973) value survey to explain the values stressed or expected by different social groups. This list includes nine values, including security, sense of belonging, sense of accomplishment, being well-respected, self-respect, self-fulfillment, fun and enjoyment of life, excitement, and warm relationships with others. Peter and Olson (1987) argue “attributes” are “means”, while “consequences” and “values” are “ends”. From a cognitive perspective, values are the psychological performance that consumers wish to achieve. Through “attributes” of products, consumers can attain a higher goal. For instance, for a segment of consumers who pay attention to the value of “health”, beverage providers can add the attribute of “lactic acid bacteria” to their product and use “promoting enterogastric peristalsis” as the main appeal of the product. By doing so, they can create a cognitive link in consumers that “lactic acid bacteria” leads to “promoting enterogastric peristalsis”, which can in turn satisfy their need for “health”.

### Consumption situations

Hansen (1972) categorizes consumption situations into deliberation situations, exposure situations, and response situations. Response situations are easier to observe compared to the other two categories of situations. Response situations can be segmented into three subcategories, including communication situations, purchase situations, and usage situations. Engel et al. (1993) further define these subcategories and clarify their respective effects on consumers’ buying decisions. Communication situations are the situations where consumers communicate with people or media. Communication with people refers to exchanging information with other people, such as salespersons, friends, and relatives. Communication with media refers to being exposed to media that stimulates buying, such as advertisements, media reports, and posters. Purchase situations are the situations where consumers are buying a product or service. Each situation consists of three elements, including the information environment, retail environment, and influence of time. Information environment refers to all the information about the product desired by the consumer. Retail environment is the store environment of the retailer. Hence, it is also called “store atmosphere”. Influence of time refers to the influence caused by time and stress. Usage situations are the situations where the purchased product is used. Products are usually used only when necessary. Hence, the environment where a product is purchased and the environment where it is used may differ.

Belk (1975) proposes five dimensions of general consumption situations, including physical surroundings, social surroundings, temporal perspective, task definition, and antecedent states. Physical surroundings refer to all visible characteristics, including the geographical location, atmosphere, and interior design. Social surroundings include people involved in the situation, their characteristics and roles, and the interactions between them. Temporal perspective is a dimension of situations which may be described by relative time or specified in units of time. For instance, the period of time from the last salary payment or last spending to the present is a temporal perspective. Task definition is an intent or requirement to select, shop for, or obtain information about a general (e.g. items for personal use) or specific purchase (e.g. a gift for someone). Antecedent states are momentary moods such as happiness, anxiety or hostility.

These situational factors affect not only consumers’ choice but also their product loyalty. The purpose of this study is to explore the consumption motivations and actual needs of consumers of ice cream specialty stores. Since customers’ motivations to buy vary across social situations, we will view different consumption situations as different social situations, including (1) on a date, (2) gathering with friends, and (3) simply craving for ice cream (Liu 2004).

## Methods

Data were collected by one-on-one interviews and the laddering method was used. Consumers may not be aware of the chain relationship between attributes, consequences, and values when making a purchase. In other words, consumers may not necessarily plan an approach to or the goal of buying beforehand. The laddering method which requires the interviewer to continuously ask further questions in response to the participant’s answer can better elicit the attributes, benefits, and values perceived by the participant.

In qualitative research, sample size is usually not as important as the appropriateness and coverage of the sample (Corbin and Strauss 2007), so a large-scale interview is not necessary at the exploratory stage. In this study, we obtained the sample using purposive sampling method from customers of cold stone stores in Taichung City. Unlike random sampling, which deliberately includes a diverse cross section of ages, backgrounds and cultures, purposive sampling concentrates on people with particular characteristics who can better assist with relevant research. Because we are researching customer value in different consumption situations (i.e., on a date, gathering with friends, craving for ice cream), we logically focus on people who visited the store for a date, people for meeting friends, and people simply for ice cream. To ensure the representativeness of the sample, it needs to collect at least 20 responses for each focused group according to Reynolds et al. (2001). More precisely, a total of 68 customers were interviewed, and 60 valid responses were obtained. These valid responses include 20 responses from customers visiting the store for a date, 20 from customers visiting the store for meeting friends, and 20 from customers visiting the store simply for ice cream. The laddering interviews with these customers were conducted during a 3-month period.

Before each interview, we explained the goal of this study and the outline of the interview to the participant. We promised anonymity of their responses and requested their consent to tape-record the interview. We also reminded them there would be no distinction between right and wrong answers, so as to increase their understanding of this study and elicit more of their inner voice. To gradually lead them into the interview, we asked them to fill out the basic data section first, which included gender, age, education degree, and occupation, and gave them an introduction to the interview. Each interview lasted for approximately 20–30 min. During the interview, we helped participants recall their consumption experiences and details on the consumption situation and constantly used questions such as “Why is this important to you” to stimulate their thinking. Besides, we also used other questions alternately (Reynolds and Gutman 1988), such as “Why do you want to eat ice cream?”, “How has it affected you?” or “What is so special about eating ice cream?”

After the interview, we performed a series of content analyses of their responses:

1. *Build categories* We classified their responses into attributes, consequences, and values based on the presence of the “from” link and the “to” link. Factors with a “from” link and without a “to” link were classified into the “values” category; factors with both a “from” link and a “to” link were classified into the “consequences” category; factors with only a “to” link were classified into the “attributes” category (Reynolds and Gutman 1988).
2. *Coding and categorization* We named each factor before coding. Researchers responsible for coding had to categorize each factor into the category it belonged to. Three researchers were invited to perform the coding. Before coding, they had received sufficient information on the research objective and related theories so as to make more accurate judgments in coding.
3. *Reliability analysis* In this study, reliability was measured by interjudge reliability. Kassarjian (1977) suggests reliability should be greater 0.80, and reliability above 0.85 is more ideal. We calculated interjudge reliability and reliability using the following equations:
  1 $${\text{Reliability}} = \frac{{{\text{n}}\, \times \,{\text{mean interjudge reliability}}}}{{\{ 1 + [(n - 1)\, \times \,{\text{mean interjudge reliability}}]\} }}$$
  2 $${\text{Interjudge reliability}} = \frac{{2{\text{M}}}}{{({\text{Ni}} + {\text{Nj}})}}$$
  where M is the number of items fully agreed by all the judges; Ni is the number of items that Judge i should have agreed; Nj is the number of items that Judge j should have agreed; n is the number of judges.

After coding and analysis, we summed the attributes, consequences, and values into an implication matrix. This matrix shows the numbers of direct and indirect links between factors and serves as the foundation for drawing hierarchical value maps (HVM). This matrix has three components. The first is the number in each column. Each number consists of an integer and a decimal. The integer denotes the number of direct links, while the decimal denotes the number of indirect links. The second component shows the in-degree and out-degree of each factor. In-degree indicates the number of times a factor is mentioned as resulting from another factor, while out-degree indicates the number of times a factor is mentioned as the source of or an antecedent to another element (Ha and Jang 2013). The third component indicates the abstractness ratio (in-degree/in-degree + out-degree). This ratio ranges between 0 and 1. A lower ratio indicates the factor is more tangible, while a higher ratio indicates the factor is more abstract (Goldenberg et al. 2000). This abstractness ratio can be the criterion for sorting factors in the implication matrix (Pieters et al. 1995).

Finally, we built HVMs based on this implication matrix. With HVMs, we can better clarify the relations between factors in the implication matrix. However, if we present all the chain relations between factors in one HVM, the HVM may be too complicated, and important links might be overshadowed. Therefore, we set a cutoff to limit the factors to include in the HVM. There is no theoretical or statistical standard for setting the cutoff. Generally, it should be set with consideration of data integrity, chart comprehensibility, and the goal of highlighting the important links between factors (Grunert and Grunert 1995). Reynolds and Gutman (1988) suggest a cutoff between 3 and 5 for a sample of 50 and 60 people. In other words, factors with a number of direct links and indirect links below 3 can be excluded from the chart. Therefore, we excluded links with a frequency below 3 and drew HVMs based on the number of links between factors.

## Results

### Basic data of the participants

The sample comprised 60 participants. In this sample, female participants constituted the majority (68 %), and most participants were in the age group of 21–30 (52 %) and single (68 %). Besides, most participants had a college or university degree (70 %). In terms of occupation, students were the largest group (52 %), followed by the service industry (22 %). As to disposable income, most participants had no more than 50,000 (28 %); the group having 5001–10,000 and the group having 10,001–20,000 comprised 20 % respectively. Participants who purchased ice cream 2–3 times a month were the largest group (48 %). Most had average spending between NT $101–200 (65 %). Among the numerous ice cream brands, Cold Stone was most frequently visited by the participants (65 %), followed by Haagen-Dazs (13 %). Taste was the major appeal to most participants (63 %). To sum up, this sample comprised mainly of female consumers, consumers visiting ice cream stores 2–3 times a month, and consumers spending $101–200 in each visit.

### Content analysis

The interview results were transcribed and semantically analyzed to produce a factor implications table. Three coders participated in this study. They categorized words and phrases in the transcription independently. Through discussion, they deleted inappropriate words and phrases, gave a name to each factor, and divided the named factors into attribute, consequence, and value tiers. For the consequence tier, the research has adopted the concept of functional consequences and psychosocial consequences proposed by Olson and Reynolds (1983). For the value tier, the research has adopted LOV as the basis of compiling customers’ terminal values. Accordingly, this research obtained 41 factors, including 14 attributes, 14 consequences, and 13 values. Table 1 shows the implications of each factor. In this study, the factor code indicates the order the factor appears in the interview. This is irrelevant to the number of times the factor is mentioned or the importance of the factor.

**Table 1:** Implications of factors

	Factor	Implications
A01	Promotions	Coupons, VIP bonus collection, and special offers
A02	Diverse products	A diversity of flavors and seasoning materials are available for choice
A033	Gathering of friends	Friends are invited to enjoy the food together
A04	Taste	The taste is pleasant, soft, fragrant, thick, and cold
A05	Service	Sampling is offered generously; employees interact with customers, sing songs, and provide water to customers
A06	Brand awareness	This brand is commonly heard; many people are eating it; everyone knows about it; it appears in ads frequently
A07	Store location	There are many stores; many are set up in department stores or near business districts
A08	Never tried it	Never tried it; first timer
A09	Appearance	The appearance of cookies, the seasoning style, delicate, elegant, looking delicious
A10	Brand word-of-mouth	Recommendation by friends
A11	Environment	Clean, the overall feeling, many seats, spacious, casual
A12	Brand image	Joyful, vigorous
A13	Ice-frying	Ice-frying process, the frying looks so real
A14	On a date	Coming with valentine
C01	Delicious	Delicious
C02	Relax	Comfortable, relaxing, and free
C03	Trust	Trust; believe; the quality is stable; guarantee
C04	Convenient	It’s just around the corner; not causing any trouble; not taking too much effort
C05	Newness	Try different flavors; I’d like to know what it tastes like
C06	Creativity	Special, new, interesting, always tasty, not monotonous, playful
C07	Good bargain	Cheap; lucky to have this good deal; the cost is minimal; cost-saving; good value for money
C08	Feeling of sharing with friends	Promoting friendship; sharing the taste; sharing calories; sharing the pleasant atmosphere
C09	Clean and tidy	Clean and tidy
C10	Tasteful	Having taste; fashionable
C11	Knowledge	Knows what it is; be confident to tell others about it
C12	Suitable for chatting	Be allowed to have a long chat; chat in loud voice
C13	Well treated	Being well treated; thoughtful; recommending flavors according to customer preference
C14	Sharing with valentine	Feeling of sharing with valentine
V01	Sense of assurance	Feeling assured; secured
V02	Efficiency	Save time for personal matters
V03	Freshness	New feeling, sense of freshness, curiosity
V04	Pleasure	Feeling good, joyful, happy
V05	Economy	I can buy more other things
V06	Satisfaction	Worthwhile, satisfactory
V07	Superiority	Feeling I am good
V08	Confidence	Confidence
V09	Health	No burden, healthy
V10	Love and belonging	It feels great to have friends; not alone
V11	Sense of ease	Relaxing, comfortable, no pressure
V12	Respect	Pay attention to customers’ voices; respect
V13	Wellness	Sense of being cared, loved

As mentioned earlier, we measured reliability by interjudge reliability. The first judge agreed with the implications of 39 factors, the second agreed with the implications of 40 factors, and the third agreed with the implications of 41 factors. According to Eqs. (1) and (2), the interjudge reliability values are 0.96, 0.98, and 0.99. The mean interjudge reliability is 0.97, and the overall reliability is 0.99, which is above the 0.85 level suggested by previous research. Therefore, the reliability of this research is assured.

### Analysis of the implication matrix

After content analysis, we obtained 161 attribute-consequence-value chains from the interview responses of 60 participants (each generating an average of 2.7 (=161/60) chains) and used these chains to build an implication matrix, as shown in Table 2. In this matrix, each number consists of an integer and a decimal, respectively denoting the frequency of direct links and the frequency of indirect links. The higher the frequency, the closer the relation between the two factors.

**Table 2:** The implication matrix for the entire sample

Code	C01	C02	C03	C04	C05	C06	C07	C08	C09	C10	C11	C12	C13	C14	V01	V02	V03	V04	V05	V06	V07	V08	V09	V10	V11	V12	V13	Total	Abstractness ratio
A01	1.0					1.0	16.0	0.1									0.1	0.2	0.15	0.1								18.20	0.000
A02					5.3	9.0								0.1			0.9	0.1		0.5							0.1	15.19	0.000
A03					0.1			4.0							0.3		0.1	2.1										6.6	0.000
A04	23.0	2.0	1.0		1.0	1.0				1.0					0.2		0.1	0.18		1.5					0.3		0.3	30.32	0.000
A05		4.1				2.0	1.0						6.1		0.1		0.1		0.1	0.1	0.2	0.1			0.4	0.4		13.17	0.000
A06			7.0		2.0			1.0		2.0	1.0				1.4		0.1				0.1	0.4	0.3					14.13	0.000
A07				7.0												0.7												7.7	0.000
A08					5.0			0.1			0.1						0.4					0.1						5.7	0.000
A09						2.0											0.1	0.1										2.2	0.000
A10			6.0												0.6													6.6	0.000
A11		5.2	0.2					0.2	2.0			4.0			0.2	0.1							0.1		0.7			11.17	0.000
A12	1.0	1.0	1.0				1.0	1.0		6.0					0.1			0.3		0.2	0.3	0.1			0.1			11.12	0.000
A13			1.0			5.0	0.1							0.1	0.1		0.4											6.6	0.000
A14																		2.0		1.0							1.1	5.1	0.000
C01														1.0	1.0			17.1		3.0					2.1		2.0	26.2	0.417
C02													1.0	1.0	0.1	1.0		1.0		1.0	1.0				10.0			15.1	0.529
C03															14.0								4.0					18.0	0.526
C04																7.0		1.0										8.0	0.467
C05								0.1			1.0						12.0	2.0		1.0		1.1						17.2	0.525
C06		1.0			3.0												8.4	1.0		5.1					0.1			18.6	0.457
C07								1.0									1.0	0.1	15.0	1.0								18.1	0.500
C08					1.0												1.1	2.1				2.0		3.0	1.0			10.2	0.538
C09			2.0												0.1								0.1					2.2	0.333
C10																		2.0		2.0	4.0	2.0						10.0	0.474
C11								1.0														1.1						2.1	0.500
C12		2.0						2.0							0.1										0.3			4.4	0.333
C13															1.0					1.0	1.0	1.0			1.0	4.0		9.0	0.471
C14																		1.0							1.0		2.0	4.0	0.500
V01																												0.0	1.000
V02																												0.0	1.000
V03																												0.0	1.000
V04																												0.0	1.000
V05																												0.0	1.000
V06																												0.0	1.000
V07																												0.0	1.000
V08																												0.0	1.000
V09																												0.0	1.000
V10																												0.0	1.000
V11																												0.0	1.000
V12																												0.0	1.000
V13																												0.0	1.000
Total	25.0	15.3	18.2	7.0	17.4	20.0	18.1	10.5	2.0	9.0	2.1	4.0	7.1	3.10	17.23	8.80	22.28	31.29	15.16	16.15	6.6	7.9	4.5	3.0	15.20	4.4	5.5		

Besides, the abstractness ratios in this matrix help distinguish between attributes, consequences, and values. Factors with a ratio of 0 are “attributes”, factors with a ratio between 0 and 1 are “consequences”, and factors with a ratio of 1 are “values”.

### Hierarchical value map (HVM)

#### The HVM for the entire sample

**Fig. 1:**
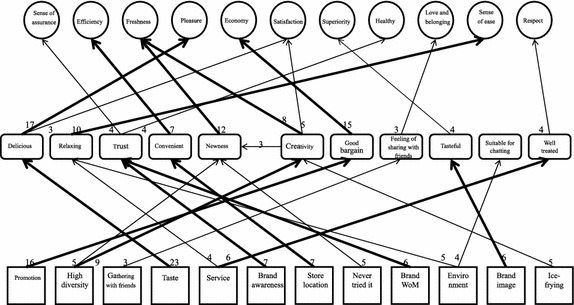
HVM for the entire sample

We set the cutoff value at 3, meaning only factors with at least 3 direct links are included in the HVM. In the HVM, each number is obtained from the integer part of the corresponding number in the implication matrix. It denotes the number of times the direct link between two factors is mentioned. The higher the number, the more important it is to the participants. To capture the major value chain among numerous value chains, we used a thick line to represent the links in the top 50 % of links ranked by frequency (i.e. 6 or more times) and a thin line to represent the other links (i.e. fewer than 6 times). The HVM is as shown in Fig. 1.

In the HVM for the entire sample, different attributes have different value chains. The thick lines show the major stable chains. These chains include “promotion-good bargain-economy”, “taste-delicious-pleasure”, and “store location-convenient-efficiency”. From these major stable chains, we can infer economy, pleasure, and efficiency are the values sought by customers when buying ice cream. Besides, customers will also consider promotional information and convenience when buying ice cream.

#### Analysis of HVMs for different consumption situations

**Fig. 2:**
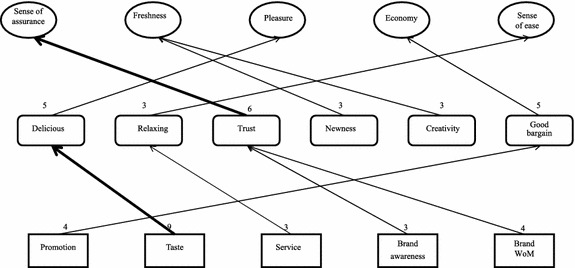
HVM for consumers on a date

**Fig. 3:**
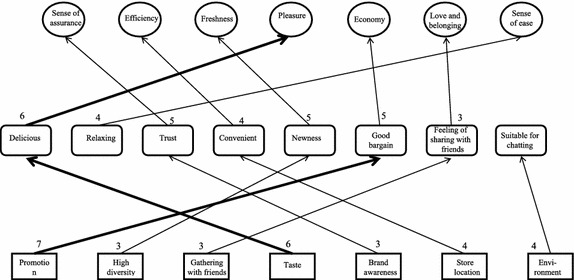
HVM for consumers gathering with friends

**Fig. 4:**
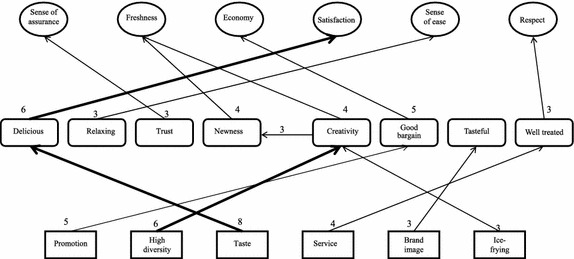
HVM for consumers visiting the store simply for ice cream

As shown in Figs. 2, 3 and 4, regardless of the situation, “promotion” always leads to the value of “economy”; “taste” leads to the value of “pleasure” for consumers on a date or gathering with friends; “taste” leads to the value of “satisfaction” for consumers simply craving for ice cream; “brand awareness” leads to “sense of assurance” for consumers in the on-a-date or gathering-with-friends situations; “high diversity” leads to “freshness” for consumers gathering with friends or simply craving for ice cream; it should be especially noted “service” leads to “sense of ease” for consumers visiting the store for a date, and “service” leads to “respect” for consumers visiting the store simply for ice cream; in the dating situation, “brand word-of-mouth” leads to “sense of assurance”; in the friends gathering situation, “gathering of friends” leads to “love and belonging” and “store location” leads to “efficiency”.

Therefore, consumers of ice cream pay attention to the economical value of promotions, regardless of the consumption situation. In the dating and friends-gathering situations, consumers are more concerned with pleasure. For these consumers, the feeling of dating with their valentine or being with their friends is more important, and so is the safety of the food. For consumers visiting the store simply for ice cream, the sense of satisfaction resulting from eating ice cream is more important.

Consumers in the on-a-date situation expect to receive relaxing services. In contrast, consumers who visit the store simply for ice cream expect special treatment from the store. As a result, they may pay more attention to the staff’s attitude and hope to receive adequate respect from them. For consumers in the on-a-date situation, brand word-of-mouth is one of the factors affecting their consumption. They wish to recommend safe food to their date and believe they can gain a sense of assurance from a store with positive word-of-mouth.

Consumers visiting the store in a friends-gathering situation pay attention to the sense of belonging. This kind of situation gives them a sense of belonging to a specific group. Besides, since they usually have a large number of friends coming to join them, store location is important. They may have reduced intention to visit stores located in a more distant area.

For consumers in a friend-gathering situation or craving-for-ice-cream situation, product diversity is important. These two groups of consumers expect to have new feeling or experiences in each purchase. A store with high product diversity allows them to try different items and gain the value of freshness in each visit.

## Conclusion and discussion

### Conclusion

In this study, we used a means-end chain approach to examine the expected values underlying in ice cream consumers in three different consumption situations. Using one-on-one interviews and laddering, we collected value perceptions from 60 ice cream consumers. From their value perceptions, we identified the important links for them and analyzed differences in the attribute-consequence-value chain between consumption situations. We discussed the customer value expected by consumers of ice cream from domestic ice cream stores and also the customer value expected by customers in different consumption situations.

We summed the attributes, consequences, and values provided by all the participants into an implication matrix. This matrix shows the numbers of direct and indirect links between factors on three levels. Based on this matrix, we further drew HVMs to more clearly illustrate the relations between factors. The HVM for the entire sample was created with a cutoff of 3. To identify important value chains, we used thick lines to indicate links in the top 50 % of links ranked by frequency (i.e. having 6 or more links). From these links, we obtained three major stable chains, including “promotion-good bargain-economy”, “taste-delicious-pleasure”, and “store location-convenience-efficiency”. These stable chains suggest “economy”, “pleasure”, and “efficiency” are key values emphasized by ice cream consumers. In addition to the pleasure of eating ice cream, consumers also pay attention to the cost-benefit ratio of the product and efficiency of the service when making a decision for ice cream consumption.

The analysis of customer value in different consumption situations shows there is no direct and stable chain in the HVM for consumers in the on-a-date situation. Though below the level of strong relation (cutoff = 6), the number of links between “taste-delicious-pleasure” is very close to this standard, suggesting the existence of an underlying chain relation. The HVM for consumers in the gathering-with-friends situation shows “taste-delicious-pleasure” is a major chain, suggesting the attribute of “taste” leads to the consequence of eating “delicious” food and ultimately to the value of “pleasure”. From this chain, we can infer consumers who visit an ice cream for gathering with friends pay more attention to the “taste” attribute of ice cream and whether they can have a pleasant feeling after visiting the store. In other words, this type of consumers expects to have a pleasant and joyful feeling in this consumption situation. The HVM for consumers visiting the store simply for ice cream shows “taste-delicious-satisfaction” is a major chain, suggesting the attribute of “taste” leads to the consequence of eating “delicious” food and ultimately to the value of “satisfaction”. From this chain, we can infer consumers who visit an ice cream store due to a craving for ice cream pay more attention to the “taste” attribute of ice cream and whether they can feel a sense of satisfaction after visiting the store. In other words, consumers in this situation expect to achieve mental satisfaction by satisfying their craving for ice cream.

### Managerial implications

#### Marketing suggestions

1. *Products* Taste is one of the most important attributes for customers. Although Cold Stone uses its US origin as a key appeal to customers, we suggest this company introduce new products based on local customers’ flavor preferences obtained through a survey and develop new or seasonal products on a regular basis. Generally, customers expect to have a diversity of product choices and be able to find one that not only meets their preference but also gives a new experience. Besides, this company can offer free sampling of their ice cream. This service allows customers to directly taste the food before they buy, so it can also help promote buying behavior.
2. *Price* Consumers expect to get the best product at the lowest cost. Therefore, price discount is an important factor affecting customers’ buying intention and behavior. Although Cold Stone launches promotional activities from time to time, many consumers do not know about these activities until the activities have expired or until they are told through word-of-mouth. Therefore, we suggest Cold Stone reinforce their marketing through social media to allow customers to access promotional information at anytime and anywhere. Besides, it can also distribute promotional leaflets in the streets near their stores to attract nearby consumers.
3. *Activities* Our findings indicate that most customers expect to gain some pleasure from eating ice cream. Cold Stone emphasizes it is an ice cream specialty store full of passion and joy, but not all customers are clear about the brand image it claims. To increase customers’ knowledge of its brand image, Cold Stone can add joyful elements to the names of its ice cream products or use celebrity appearances in its advertisements to underscore the joyful feeling its products can offer. Besides, it can also use themed parties to create a venue full of joy and imagination for customers. This marketing approach may deepen customers’ impression of surprises and happiness offered by the company.
4. *Store location* Ice cream is not a daily necessity. Customers usually have low intention to travel a long way to buy ice cream. Therefore, when setting up a new store, Cold Stone can consider places in an important business district or places with a convenient parking area nearby, such as department stores, wholesale stores, parks, and so on. Stores set in these kinds of places are more easily accessible to customers. Besides, stores located in places with more people coming and going can attract more consumers who have a craving for ice cream.

#### Suggestions on market segmentation

The results show the underlying major link for the on-a-date situation is the same as the major link for the gathering-with-friends situation. Therefore, we view customers in these two consumption situations as in the same market segment. Below are our suggestions:

1. *The* “*on-a-date*” *situation and the* “*gathering-with-friends*” *situation* For customers in either situation, “taste” leads to “pleasure”. This indicates customers in these situations expect to gain the value of pleasure in the consumption process. Therefore, in addition to incorporating joyful elements into their products and advertisements, Cold Stone can provide set meals for two (or more) and interactive games to amplify customers’ feeling of sharing and pleasure in these situations.
2. *The* “*craving for ice cream*” *situation* For customers in this situation, “taste” leads to “satisfaction”. This indicates customers in this situation expect to gain the value of satisfaction in the consumption process. This type of customer is more concerned with satisfaction of their taste. Therefore, while increasing the diversity of products, Cold Stone can provide a description of each product item and offer the free sampling service to help customers understand the content (e.g. raspberry, chocolate) and flavor (e.g. sour, sweet, low-fat) of each item. These approaches are helpful for customers to pick the right item for themselves and then gain the value of satisfaction.

### Limitations and suggestions

The limitations of this study and suggestions for future researchers are as follows: (1) include multiple ice cream brands in the survey: due to time and human resource constraints, we only selected Cold Stone stores in Taichung City as our subjects. Future researchers are advised to conduct a cross-region study of several ice cream brands. This kind of study may produce more comprehensive findings about ice cream consumers’ value perceptions. (2) Include other variables in the analysis: in addition to a general analysis of customer value among customers of ice cream stores, we also analyzed customer value in different consumption situations. However, future researchers can include variables, such as demographic variables or family life cycle, in the analysis. (3) Use a large sample: we used soft laddering in the interviews with customers to well know the researched area. Future researchers can use hard laddering with more structured interview and data collection procedure, making it possible to collect more samples in minimizing a researcher’s influence.
